# Dismantling task-sharing psychosocial interventions to personalize care for people affected by common mental disorders: developing a taxonomy of active ingredients and ranking their efficacy

**DOI:** 10.1192/j.eurpsy.2024.1390

**Published:** 2024-08-27

**Authors:** D. Papola, V. Patel, C. Barbui

**Affiliations:** ^1^Global Health and Social Medicine, Harvard Medical School, Boston, United States; ^2^Neuroscience, Biomedicine and Movement Sciences, University of Verona, Verona, Italy

## Abstract

**Introduction:**

The global burden associated with common mental disorders is high, especially for people living in low resource settings. Although psychosocial interventions delivered by locally available lay or community health workers are effective, mechanisms of intervention response are poorly understood. One of the greatest barriers is that psychosocial interventions are administered as complex, multi-component “packages of care”.

**Objectives:**

Our aim is to systematically review all the randomized controlled trials (RCTs) that have tested the efficacy of psychosocial interventions delivered through the task shifting modality to treat people suffering from common mental disorders (depression, anxiety, and related somatic complaints) in low resource settings, dismantle the intervention protocols creating a taxonomy of active intervention components, and re-evaluate their efficacy.

**Methods:**

We will use the component network meta-analysis (cNMA) methodology. The major benefit of cNMA is the possibility to disentangle intervention components and explore their effectiveness separately or in various combinations (even in disconnected networks). cNMA increases statistical power by combining direct and indirect comparisons while fully respecting the randomized structure of the evidence. According to the additive cNMA model which we will implement, adding a component “c” to a composite intervention “X” will lead to an increase (or decrease) of the effect size by an amount only dependent on “c”, and not on “X”. We will denote the corresponding component specific incremental standard mean difference (iSMD) so that iSMDc = SMD(X+c) v. (X). Combining these component-specific iSMDs will allow the estimation of SMD between any two composite interventions.

**Results:**

A network of comparisons and a hierarchy that includes all intervention components expressed as iSMD, indicating the added benefit of adding a component to an intervention, will be presented. By selecting the most effective components it will be possible to outline a novel task shifting psychosocial intervention to be tested in future RCTs.

**Conclusions:**

These findings will set the basis for further investigations in the field of precision medicine. This project is funded by the European Union’s HORIZON EUROPE research programme under grant agreement No 101061648.

**Disclosure of Interest:**

None Declared

